# Improvement of the aerodynamic behaviour of the passenger car by using a combine of ditch and base bleed

**DOI:** 10.1038/s41598-022-23183-z

**Published:** 2022-11-02

**Authors:** Ahmed Al-Saadi, Khaled Al-Farhany, Ahmed Essa Faisal, Mohammed Azeez Alomari, Wasim Jamshed, Mohamed R. Eid, El Sayed M. Tag El Din, Ayesha Amjad

**Affiliations:** 1 Department of Mechanical Engineering, University of Al-Qadisiyah, Al-Qadisiyah, 58001 Iraq; 2 Department of Mathematics, Capital University of Science and Technology (CUST), Islamabad, 44000 Pakistan; 3 Department of Mathematics, Faculty of Science, New Valley University, Al-Kharga, 72511 Al-Wadi Al-Gadid Egypt; 4 Department of Mathematics, Faculty of Science, Northern Border University, Arar, 1321 Saudi Arabia; 5 Electrical Engineering, Faculty of Engineering and Technology, Future University in Egypt, New Cairo, 11835 Egypt; 6 Faculty of Organization and Management, Silesian University of Technology, 44-100 Gliwice, Poland

**Keywords:** Mathematics and computing, Physics

## Abstract

The current study investigates different methods to minimize the drag coefficient (*C*_*D*_) without ignoring the safety factor related to the stability of a vehicle, i.e., the lift coefficient (*C*_*L*_). The study was carried out by employing an SUV car analyzed numerically using one of the CFD software, Ansys. Four different models such as realizable *k–ε*, standard *k–ω*, shear stress transport *k–ω*, and Reynolds stress model (RSM). The considered models have been validated with experimental data and found in good agreement. The considered inlet velocity varies from 28 to 40 m/s, the results showed that the drag coefficient and the stability are both improved by applying a modification on the roof of the considered car.

## Introduction

In today’s society, road vehicles’ safety, effectiveness, and convenience are critical characteristics. At the same time, low fuel usage is critical for emerging countries since it helps with economic growth. The majority of cars in developing countries are petroleum-powered, and fuel consumption is rising at an alarming rate of 5% per year. As a result, experts are attempting to lower the fuel consumption of road vehicles; even a slight reduction will be a significant accomplishment^1–3^. Traditional methods of lowering a vehicle’s fuel consumption include reducing its total weight, adjusting the engine volume, the engine combustion process, and so on, all of which directly impact its comfort and performance. Aerodynamic experiments on a hatchback automobile were carried out to improve both characteristics at the same time. The hatchback automobile was picked because it is favored by most individuals who value their money^4–7^. The global demand for fuel, as well as the environmental issues, push researchers to investigate ways to minimize the consumption of fuel. Furthermore, the vehicles have been registered as the major fuel consumers. For that reason, the researchers have done an extensive study to minimize fuel consumption at the same performance. They found that this goal can be achieved by reducing the aerodynamic drag; hence, the required performance can be optioned with less fuel consumption^8–11^. Furthermore, global warming increases the pressure on designers to make efforts to design vehicles with low drag coefficients using minimal changes in the shape Krishnani et al.^12^. A wagon car can be considered a good example that has a relatively high rake angle ($\phi$), producing the separation before the back windscreen. The increase of the flow turbulence in the wake causes ${C}_{D}$ to increase and hence higher consumption of fuel. So, to decrease the turbulence, there should be a delay in the flow separation. A high rake angle reduces the downwash and the induced drag but will have a negative impact on the pressure drag Levin et al.^13^. Guo et al.^14^ conducted a numerical investigation, exploding $k$–epsilon model, on the wagon car to modify the front surface while the car’s bottom was assumed to be flat. Furthermore, the authors neglected the effect of mirrors, wheels, and wind gaps using the FEM. They stated that an increase of the slantwise angle causes an increase in the air resistance and hence decreases the lifting force. Barbut and Negrus^15^ carried out a numerical study on sedan vehicles to understand how the air was affected by the design of the lower part. This study employed the DxUNSp code, and the considered length of the car was $1.5\mathrm{ m}$. The main results showed that the DC could be reduced to around 20% by modifying the lower part design. Song et al.^16^ modified a sedan vehicle’s outer design by applying the ANN method. The main focus of this paper was on the rear shapes of the car’s body in order to reduce ${C}_{D}$. The main variables that have been considered were six points that were chosen from the car’s end. They stated that fuel consumption efficiency can be improved by making some acceptable modifications to the back of the car. Koike et al.^17^ investigated, numerically, the effect of adding vortex generators (VGs) on the sedan car in order to reduce the ${C}_{D}$ and hence reduce fuel consumption. They found that installing the VGs at the car’s rear part can delay the flow’s separation and hence reduce the turbulence wake. Hu and Wong carried out a numerical investigation on salon cars to study the effect of installing spoilers on fuel consumption. The results showed that the spoiler could minimize fuel consumption by a noticeable percentage. Kang et al.^18^ investigated the effect of installing a semi diffuser positioned on the end bumper of a sedan car on the reduction of aerodynamics. The authors stated that the improvements that they added to reduce the ${C}_{D}$ is only active when the speed exceeded 70 $\mathrm{ km}/\mathrm{h}$. Sivaraj and Raj^19^ investigated the aerodynamic reduction by adding the base bleed, and the results stated an improvement in ${C}_{D}$ reduction as well as fuel consumption. Different turbulence models were evaluated in earlier investigations, and the contrasts between them were examined. Based on the circumstances of a case, these investigations can assist researchers in selecting an appropriate turbulence model^20–22^. According to earlier research^23–25^, *k*–$\varepsilon$ turbulence model might be employing to recreate the aerodynamics of automobiles.

The present examination used the same technique on a standard passenger automobile to improve fuel performance and efficiency. When the vehicle moves, there is contact between the air movement and the car’s surface, influencing energy consumption, performance, and vehicle stability. As a result, aerodynamic design evaluates how airflow regulates the cooling of the engine, gearbox, brake, and condenser. Meanwhile, boosting directional and crosswind stability while the vehicle is traveling at high speeds. It improves passenger comfort by providing ventilation, and conditioning systems, reducing dirt and mud deposition on the vehicle, and reducing wind noise. The current study investigated an improvement to reduce the consumption of fuel by minimizing ${C}_{D}$ without ignoring the importance of vehicle stability. The study employed the Land Rover Discovery^26^ as a sample which has been tested numerically using Ansys software.

## Numerical simulation method

### Vehicle models

**Figure 1:**
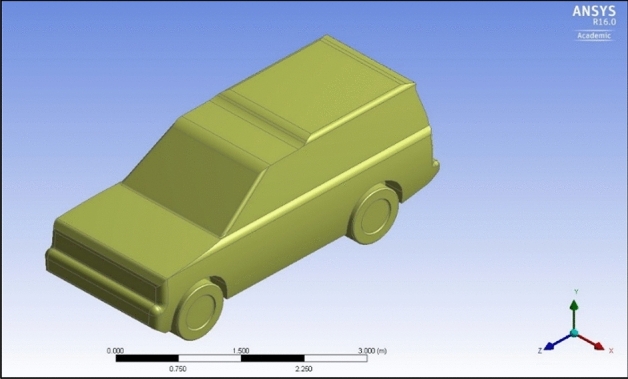
The original design of LR Discovery.

The numerical analysis of the current research is carried out using the Ansys. The input dimensions for the considered sample, LR discovery are $4.835\mathrm{ m}$, $1.887 \, \mathrm{ m}$, $1.915 \, \mathrm{ m}$, and $2.51 \, \mathrm{ m}$ for the length, height, width without mirrors, and the wheelbase, respectively, as can be seen in Fig. 1, which demonstrates 3-D of the considered sample.

**Figure 2:**
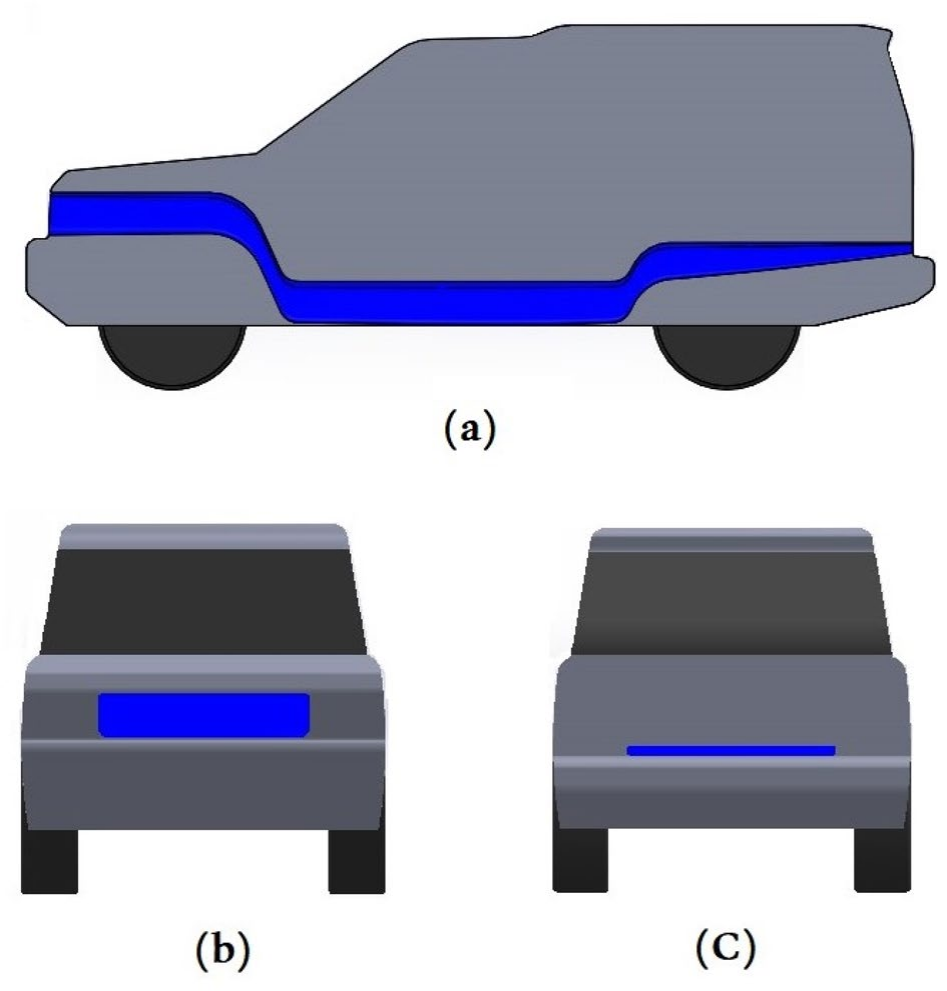
Shows the ventilation duct (**a**) the side cross-sectional area, (**b**) the front view, (**c**) the back view.

This study proposed different models to improve the downforce and reduce the ${C}_{D}$. The ventilation duct, one of the considered modifications along the LR vehicle, can be seen in Fig. 2 in blue color. This modified model has the same frontal area as the baseline model, which is 3.144 m^2^. The benefits of this modification lead to reducing ${C}_{D}$, reducing the vortices behind the vehicle, and cooling the engine. The design of the duct has been done carefully in order to avoid the capacity effect; for that reason, the dimensions of the duct are changed along with the car. The size of the duct is $0.228 \, {\mathrm{m}}^{2}$ (1.11 m × 0.205 m) at the inlet then decreases to be $0.099 \, {\mathrm{m}}^{2}$ (0.492 m × 0.203 m) at the middle and about $0.06 \, {\mathrm{m}}^{2}$ (1.11 m × 0.054 m) at the end of the car.

**Figure 3:**
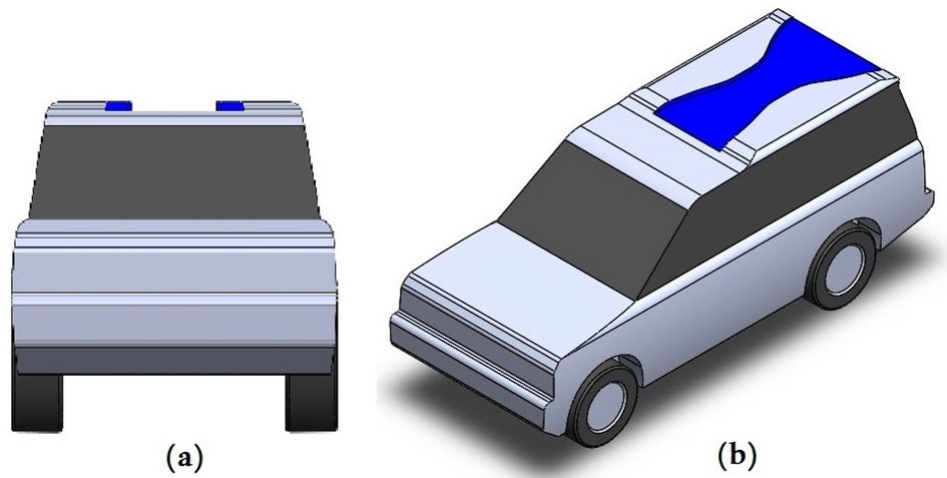
LR Discovery model with the modified roof (**a**) front view and (**b**) ISO view.

The second modification, which is tested in this paper, is shown in Fig. 3 in blue color, this technique is important for the stability of the vehicle by increasing the pressure on the roof to improve the aerodynamic behavior. The dimensions of the roof modification have been chosen based on the optimum conditions of the car design and anticipated aerodynamic behavior. The width of roof modification at the beginning of the main roof is $0.832\mathrm{ m}$ then decreases to $0.5\mathrm{ m}$ and becomes $1.183\mathrm{ m}$ at the end of the roof. The depth of this modification is $50\mathrm{ mm}$.

The ditch on the roof model has a frontal area less than the baseline model by $0.025 {\mathrm{m}}^{2}$ ($500\mathrm{ mm}\times 50\mathrm{ mm}$), which means 1/125.76 because the frontal area of the baseline model is $3.011 {\mathrm{m}}^{2}$ while for the ditch model, it is $2.986 {\mathrm{m}}^{2}$. Therefore, we can neglect the reduction in the area to compare the baseline model with the ditch model. The following figures illustrate the front views of these two models.

Another modification is a combination of the two previous modifications. These modifications could potentially generate a lower drag force and higher downforce than the baseline model.

### Computational domain

**Figure 4:**
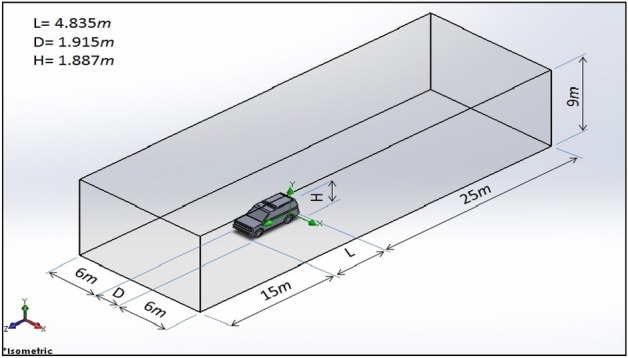
The computational domain with dimensions.

The dimensions of the computational domain have been chosen precisely to avoid the possible effects of the walls, and the dimensions are $44.835\mathrm{ m}$, $9\mathrm{ m}$, and $13.915\mathrm{ m}$ for the length, high, and width. Furthermore, the dimensions of the care are $4.835\mathrm{ m}$, $1.915\mathrm{ m}$, and $1.887\mathrm{ m}$ for the length, high, and width; consequently, the dimensions of the compactional domain are larger enough to get rid of the effect of the walls and to capture the important physics, especially behind the car where the vortices take large area as can be seen in Fig. 4.

**Figure 5:**
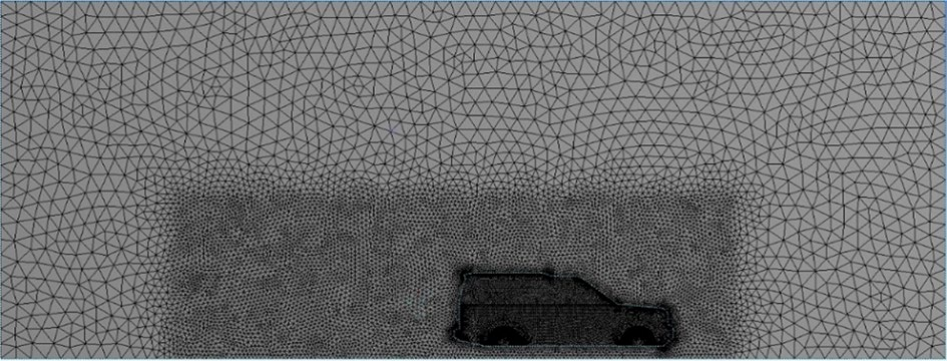
Mesh with one volume control box (VCB) around the car.

**Figure 6:**
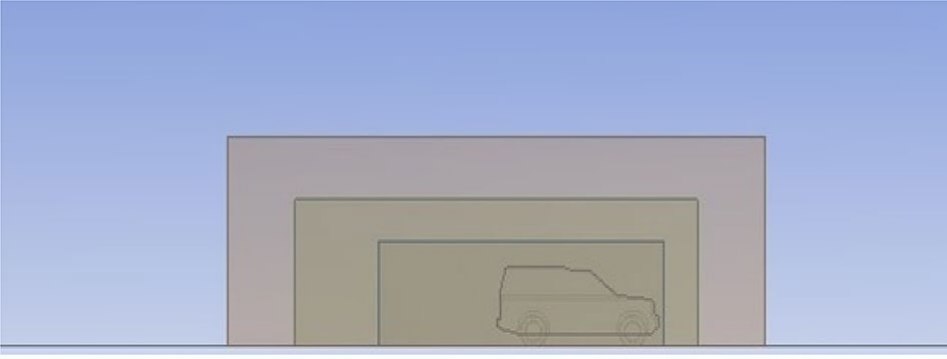
Three VCBs around the car.

**Figure 7:**
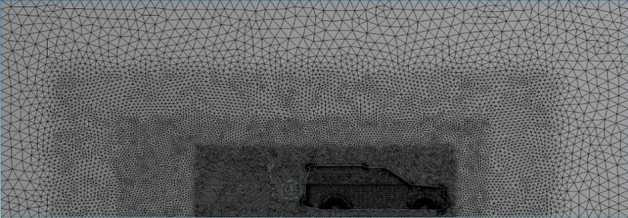
Mesh with three VCBs around the car.

**Figure 8:**
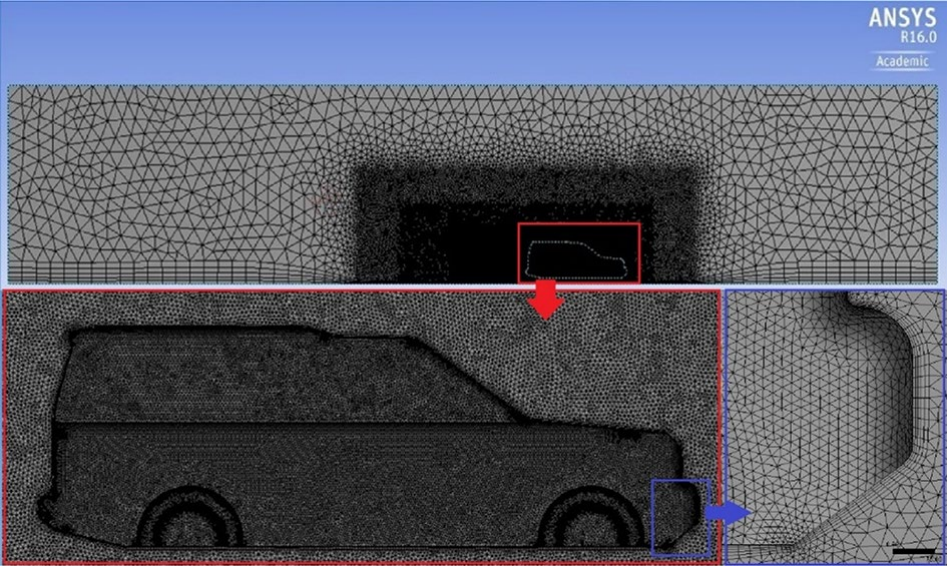
Five inflation layers mesh around the car and over the road.

### Numerical grids

The accuracy of the simulation relay on the quality of meshing. The computational domain has been divided into three layers depending on the variation of physics: the region close to the car, which includes a huge physics variation, needs to be smooth and fine enough to capture the physics. As we move away from the car, the physics variation becomes less, so grid cells become a bit larger at layer 2. As we move further, the physics variation becomes even less and the mesh required to capture physics in this region becomes coarser, as seen in Figs. 5, 6, 7 and 8. Meshing is a crucially important step in design and analysis, and this step has been done using Ansys.

**Figure 9:**
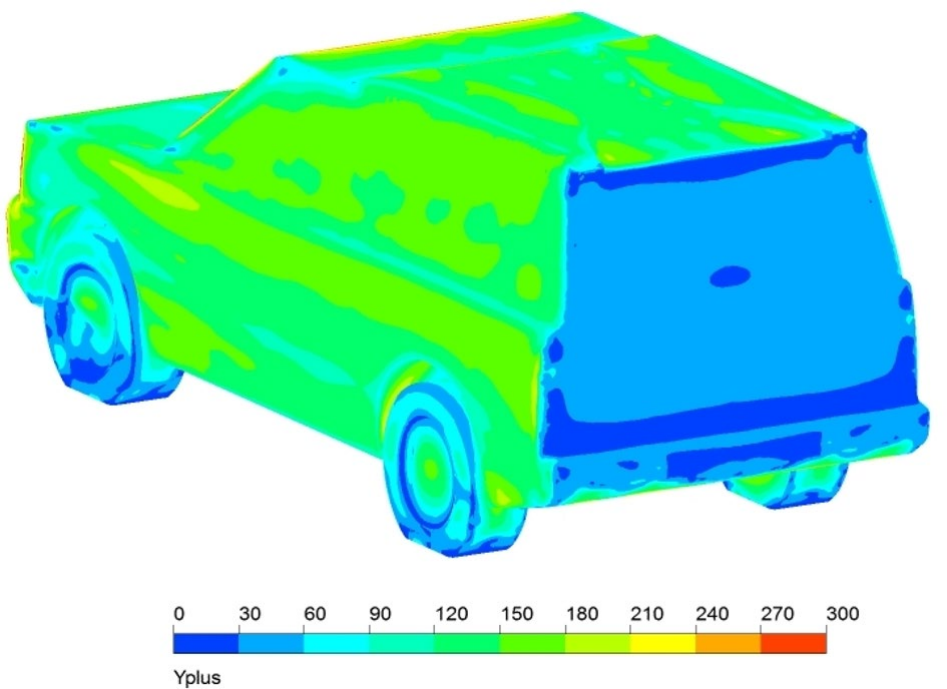
y^+^ on the modified roof model surface.

It is important to check mesh dependency before the analysis in order to choose a lower number of cells at which the results become independent of the cell change. This technique is very important to have accurate results with less time-consuming. An extensive study to choose the appropriate number of elements is achieved, which was 13 × 10^6^ elements. Furthermore, the maximum Skewness of standard mesh was 0.897066, and the minimum orthogonal quality was 0.012722. y^+^ for each configuration of Discovery car has a verity range of magnitude depending on the location. Figure 9 illustrates y^+^ for the modified roof model of Discovery car. Using a turbulence model with a wall function is the best choice regarding these numbers of y^+^.

### Boundary conditions

In this work, from the front side of the computational domain, uniform inflow speeds of $28$, $34$, and $40\mathrm{ m}/\mathrm{s}$ were utilized. The density of air ($\rho$) is $1.225\mathrm{ kg}/{\mathrm{m}}^{3}$. Reynolds numbers ($Re$) of the system were 9 × 10^6^, 11 × 10^6^, and 13 × 10^6^ for the aforementioned velocities, respectively. Experimental tests for the baseline model of the Discovery car were done in a MIRA wind tunnel in the UK^26,27^. Discovery car and all its tires were stationary inside the MIRA wind tunnel. The stationary road under the vehicle is used in numerical simulation, as is the experimental test conducted by MIRA wind tunnel. The inlet velocity of airflow was about 28 m/s at the inlet section of a wind tunnel. In the experimental test, turbulence intensity was 2.65% at t the inlet of the MIRA wind tunnel. The same turbulence intensity is used in all numerical simulations. Two types of wall boundary conditions for the sidewalls and top wall of the computational domain could have been: (i) non-slip and (ii) stationary walls. As a result, there was no vicious influence on the analyses because the domain’s external walls were identical. The car’s tyres have all been stationary, analogous to the wind tunnel model. To optimize the design and mesh, the underbody surface became flat.

### Discretization and numerical setup

The second-order upwind approach has been used for the momentum, turbulent kinetic energy, and turbulent dissipation rate. Also, in terms of spatial discretization, it was used for pressure. The relaxation factor was set to 0.25. The four turbulence models adopted for this research were realizable *k*–$\varepsilon$, standard *k*–$\omega$, shear stress transport *k*–$\omega$ (SST), and a Reynolds stress model (RSM). Most previous researchers have been using these models to examine automobile performance of vehicles, and they would result in an acceptable processing time. In this study, the two formulas mentioned below are applied to calculate the drag and lift coefficients:

$${C}_{D}=\frac{{F}_{D}}{\left(\rho {V}^{2} A\right)/2}$$

$${C}_{L}=\frac{{F}_{L}}{\left(\rho {V}^{2} A\right)/2}$$

${F}_{D}$ represents the drag force ($\mathrm{N}$), ${F}_{L}$ represents the lift force ($\mathrm{N}$), $\rho$ is the density of air ($\mathrm{kg}/{\mathrm{m}}^{3}$), $V$ represents the initial air velocity ($\mathrm{m}/\mathrm{s}$), and A represents the car’s frontal cross-sectional area (${\mathrm{m}}^{2}$).

### Car model setup

ANSYS 16.0 was used to simulate the full-scale model mentioned above. The solution domain, created within ANSYS Meshing (Version 16.0), consisted of the global domain and three VCBs around the car. Everywhere in the domain, irregular tetrahedral cells have been employed. The velocity profiles around the car’s surfaces were accurately estimated using five inflation layers with prismatic cells. The real model of the automobile and the model used in the simulation have various variations. Side mirrors, revolving wheels, and a series of complicated geometric pieces under the car are all present in a real car. The tyres on the simulation model were entirely fixed, just as in the wind tunnel model. To reduce the geometry as well as the mesh, a flat surface was utilized for the simulation model. Presumptions for real, experimental, and numerical models are listed in Table 1.

**Table 1:** The assumptions of the main conditions.

	Car models		
	Real	Experimental	Numerical
The flat surface on the underbody side	No	No	Yes
Wheel rotation	Yes	No	No
Two side mirrors	Yes	Yes	No
Engine runs and rejects exhaust gases	Yes	No	No
The center of mass is constant	No	Yes	Yes
All parts of the vehicle are from the same material	No	No	Yes
Clearance between the underbody of the vehicle and the road is constant	No	Yes	Yes
Some air in front of the vehicle enters to engine	Yes	No	No

## Results

**Figure 10:**
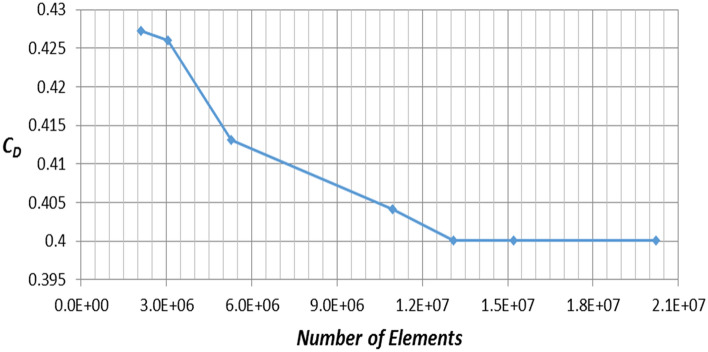
Grid independence for realizable *k*–$\varepsilon$ model.

**Figure 11:**
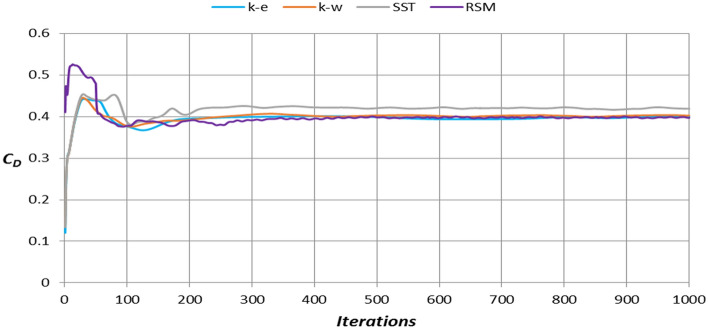
The drag coefficient for the baseline model uses four types of turbulence models.

This section describes the results of the numerical simulations of the land rover Discovery models. Because of the system’s symmetry, half of the computational domain and geometry were used to cut down on computation time. The Land Rover Discovery models were generated by using four distinct turbulence models. The sensitivity analysis obtained the optimum number of mesh elements as that could significantly affect the results. Figure 10 depicts a grid dependency analysis for the realizable *k–ε* model. The mesh number affects the drag coefficient estimate until approximately 13 × 10^6^ elements are used for 50% of the computational domain and geometry, after which there is no mesh dependency. With these simulations, the mesh elements are between 13 × 10^6^ and 15 × 10^6^. Four different turbulence models have been used to choose the best model for this investigation. Figure 11 illustrates the drag coefficient for Land Rover Discovery’s baseline model utilizing four different turbulence models. Realizable *k*–$\varepsilon$, standard *k*–$\omega$, and Reynolds stress model drag coefficients are all near the experimental data. It is 0.4, although the result of SST *k*–$\omega$ is considerably consistent with the experimental data. Because this form of turbulence model does not employ wall functions, the y^+^ value is a crucial parameter for the SST model. In this study, y^+^ was 5, which is just acceptable. Using a turbulence model with wall function (*k–ε* turbulence model) is the best choice in case of y^+^ is not low enough to obtain accurate results. Realizable $k-\varepsilon$ is suitable for high Reynolds number cases. As shown in Fig. 11, the computed drag coefficient does not change significantly after 400 iterations.

**Figure 12:**
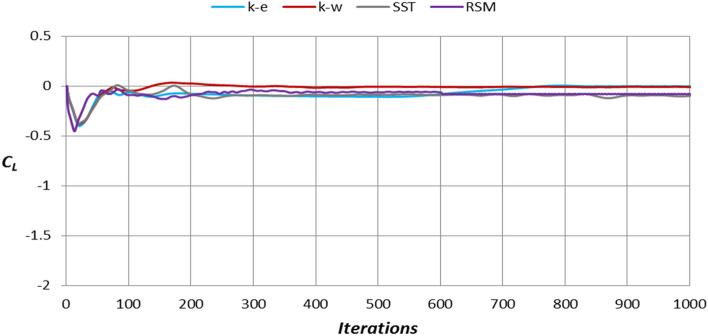
Lift coefficient for the baseline model using four types of turbulence models.

Figure 12 depicts the lift coefficient for the baseline model using four types of turbulence models. Similarly, the lift coefficient of these four types of turbulence models does not vary significantly after 400 iterations.

Table 2 compares the drag coefficient derived by modeling for the baseline model to experimental data acquired from the Land Rover Company’s official website (The official Media Centre for Jaguar Land Rover, 2012).

**Table 2:** Validation of numerical results.

Experimental result $[0, 0]$	Numerical results			
	Realizable *k*–$\varepsilon$	Standard *k*–$\omega$	Shear stress transport	Reynolds stress model
${C}_{D}=0.4$	${C}_{D}=0.4$	${C}_{D}=0.405$	${C}_{D}=0.418$	${C}_{D}=0.397$
Difference with the experimental measurement (%)	0.028	1.237	4.485	0.747

All turbulence models fit the experimental data well, but the realizable *k*–$\varepsilon$ values are the best. Realizable $k-\varepsilon$ is suited for complicated geometries and shear layers in outer flows. The kinetic energy ($k$) and turbulent dissipation ($\varepsilon$) are predetermined in this model.

**Figure 13:**
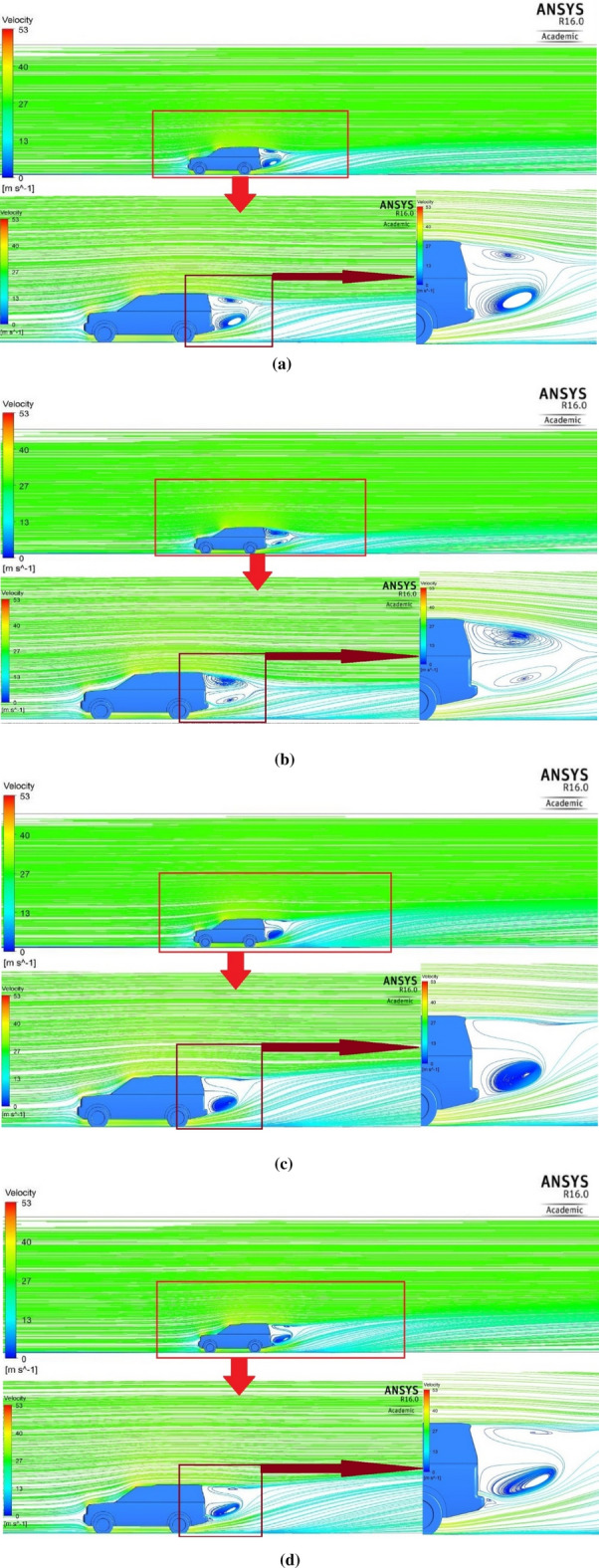
**(a)** Streamline around the baseline of the Land Rover Discovery. **(b)** Streamline around the Land Rover Discovery with the ventilation duct. **(c)** Streamline around the Land Rover Discovery with roof modification. **(d)** Streamline around the Land Rover Discovery with the ventilation duct and roof modification.

Figure 13 shows the streamline around the car for the initial air velocity at $28\mathrm{ m}/\mathrm{s}$ and with two closed-up views. The flow flowing down the roof edge and the flow from beneath the automobile body create two straight spanwise vortices. The minimum velocity is $0\mathrm{ m}/\mathrm{s},$ and the greatest velocity is $53\mathrm{ m}/\mathrm{s}$. This design has two significant issues: vortices in the region behind the car and excessive airspeed in front of the hood and roof. Due to low pressure in the rear bumper region and after the roof’s end flow separation, the vortices are non-uniform (dark blue color). The vortices can be minimized and become more uniform by adding the ventilation duct to the baseline model, as shown in Fig. 13b. The ventilation duct works to decrease the static pressure in the front of the car while increasing it behind the vehicle. The air comes out of the duct outlet and moves down because the static pressure of air behind the back bumper is less than it is at the end of the duct outlet. As a result of the pressure at the duct, the exit is higher than the pressure near the top, causing the vortex near the end of the roof to grow. The peak air velocity for a Land Rover Discovery equipped with a ventilation duct is less than that of the baseline model because some air goes through the ventilation duct. It should be noted that the duct was not straight to avoid the effect on the vehicle’s capacity and comfort. Figure 13c depicts the streamline all over the Land Rover Discovery with only the roof adjusted (without the duct). By altering the vehicle’s roof, the vortexes move from the zone near the roof’s edge to the zone near the car’s back door. The roof has been modified into a shape comparable to a convergent-divergent nozzle to raise the static pressure at the roof’s end. The Land Rover Discovery’s new exterior design, complete with all the alterations (duct and roof modification) as shown in Fig. 13d, generates the most uniform streamline. This model has a lower drag coefficient than the baseline model but higher than the model with the ventilation duct. As a result, the static pressure at the roof’s end is higher than the static pressure at the duct outlet. There is a swirl around the duct outlet in the combined model.

**Figure 14:**
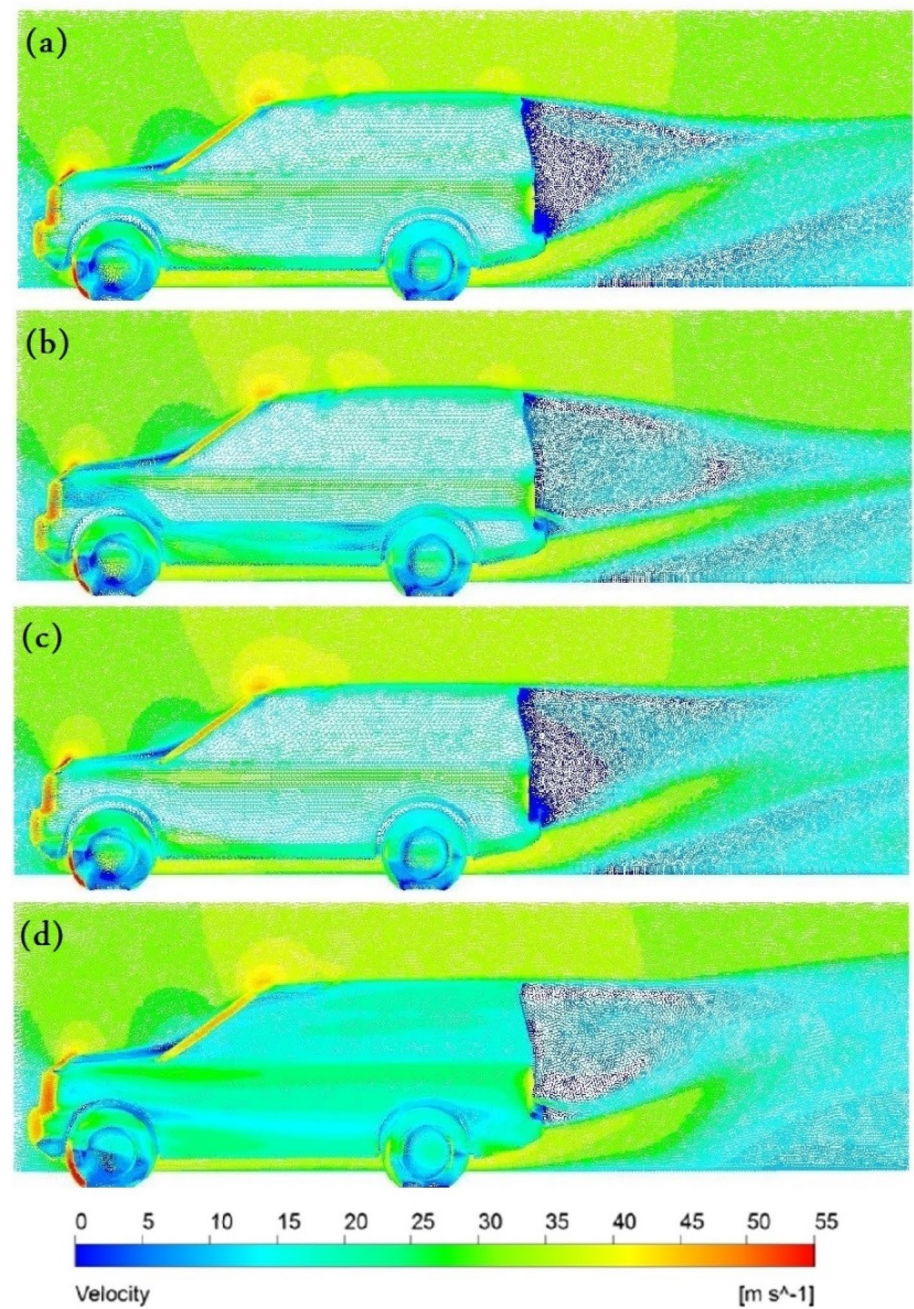
Velocity vectors for (**a**) the baseline model, (**b**) the ventilation duct, (**c**) the roof modified model, (**d**) modified combined model.

Figure 14 depicts the vehicle’s velocity vectors on all sides as well as all volume control boxes for the initial air velocity at $28\mathrm{ m}/\mathrm{s}$. Figure 14a shows the baseline model with high velocity in front of the bonnet, A-pillars, sidelights, and the front of the roof. Two very low-pressure areas are behind the car: the rear windscreen and the low part of the back door. The wake zone is more than 2 m long, and the air velocity under the automobile is around 34 m per second. The injection of air behind the vehicle After adding a ventilation duct as indicated in Fig. 14b, the maximum velocity drops, and the pressure behind the car rise. Also, the figure presents the wake region as being longer in the duct model, and the velocity vectors have a more uniform dispersion than the baseline pattern. Because a portion of the airflow through the duct is reduced, all air velocities at the automobile’s front are reduced. The velocity vectors on the bonnet are more uniform than the baseline model. The vortices inside the wheelhouses and around the tyres of the car are less than those of the baseline model. Adjusting the roof raises velocity vectors’ distribution at the wake’s top limit. Still, everything else remains the same as in the baseline model, as seen in Fig. 14c. The combined ventilation duct and roof modification velocity vectors are presented in Fig. 14d. The length of the wake zone is less than other models, but there are a lot of very low-velocity vectors, especially around the wake zone. This modification provides the best velocity distribution in the car’s wheelhouses and around the car’s tyres.

**Figure 15:**
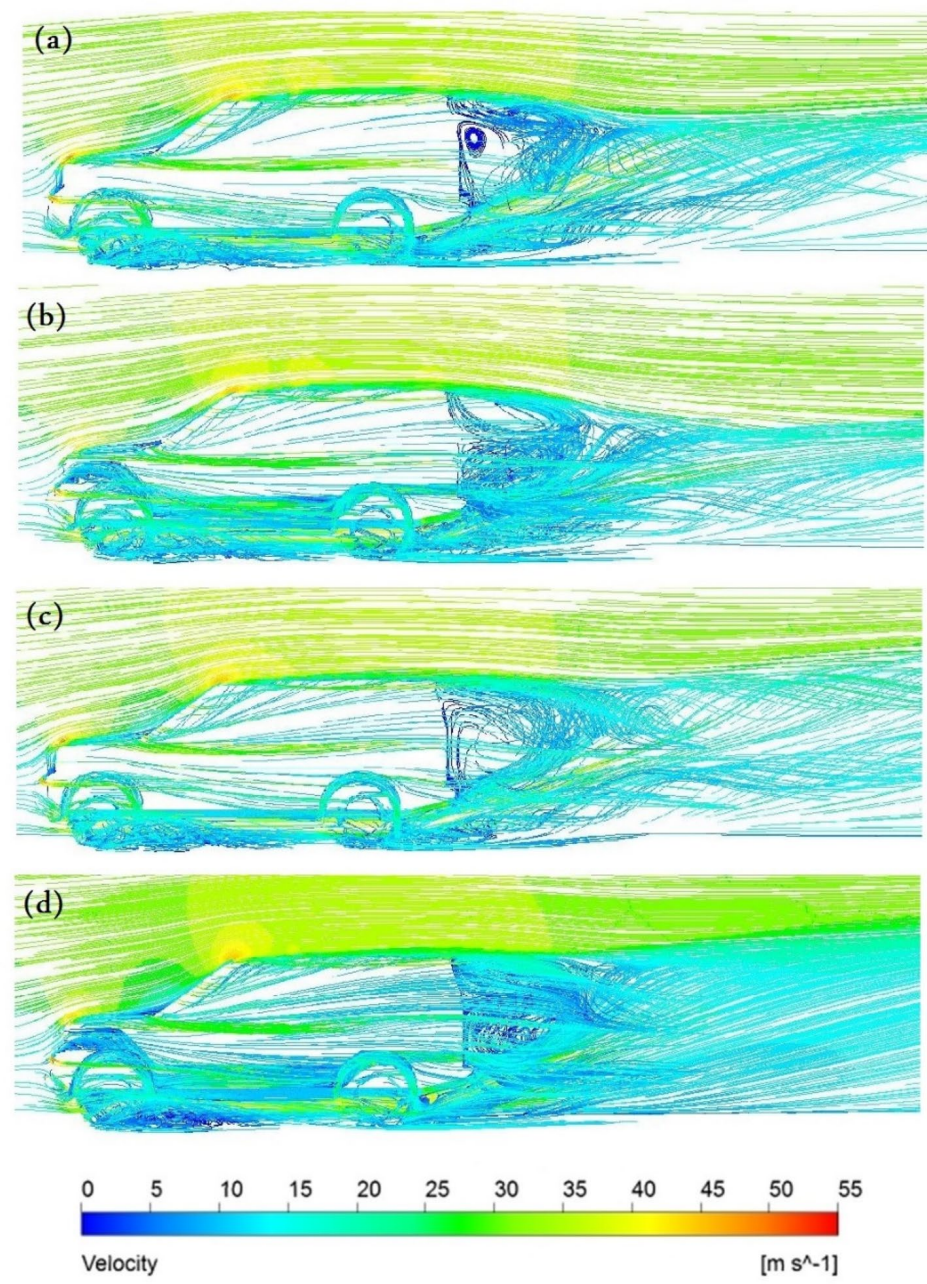
Pathlines (**a**) the baseline model, (**b**) the ventilation duct, (**c**) the roof modified model, (**d**) modified combined model.

**Figure 16:**
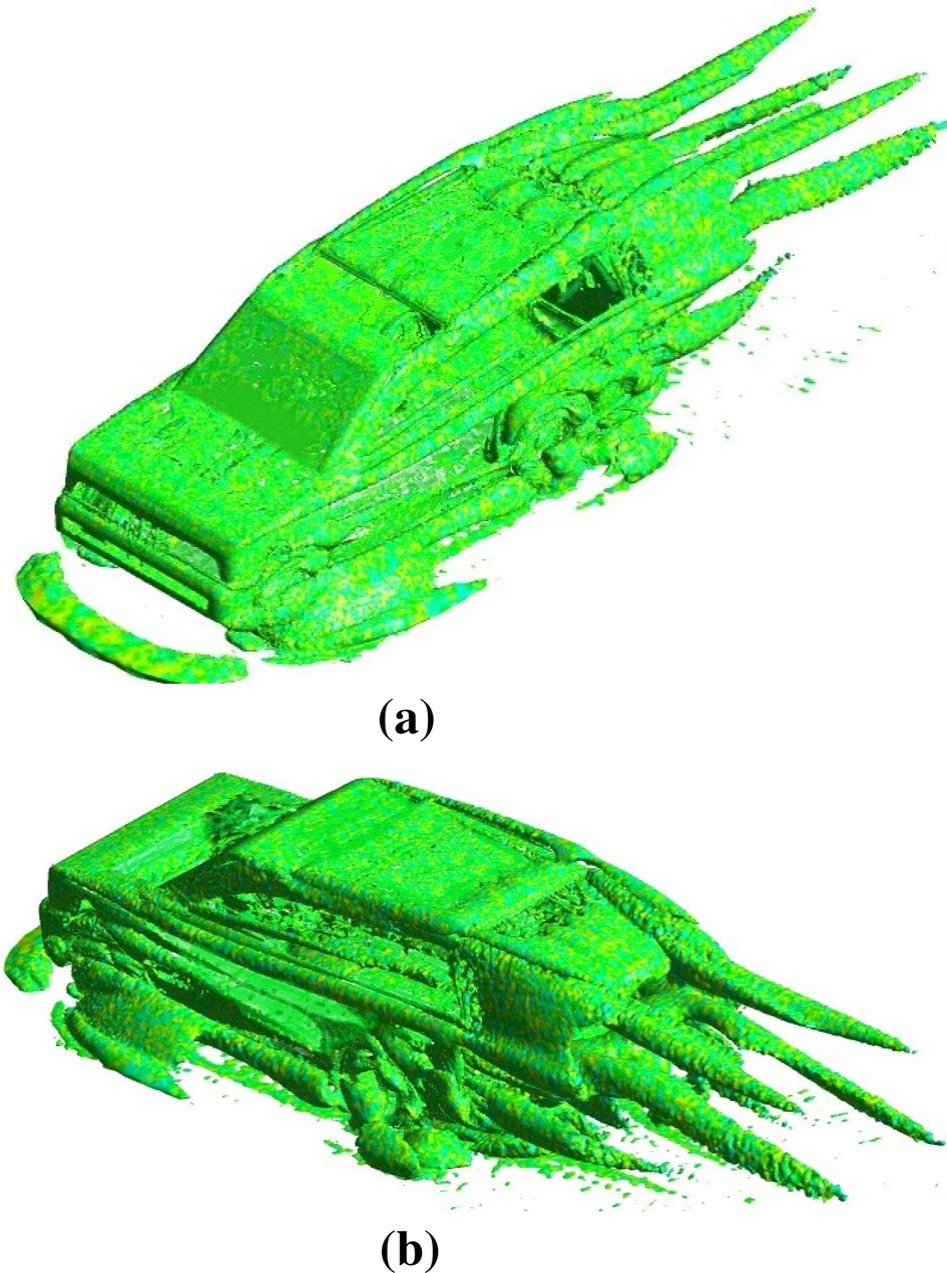
**(a)** Vortices around the baseline Discovery car from a front angle view. **(b)**. Vortices around the baseline Discovery car from a back angle view.

Figure 15 depicts the magnitude velocity pathlines for all models at an initial air velocity of 28 m/s. The wake region is more than $2\mathrm{ m }$ for the baseline model with many vortices behind the car, especially near the car's rear windscreen, as The Land Rover Discovery’s back design is comparable to a box form, shown in Fig. 15a. The ventilation duct works as passive ventilation, which means some air injecting behind the car, as shown in Fig. 15b. The distribution of the air behind the car helps to eliminate the vortices. The SUV variant with a ventilation duct has a more uniform aerodynamic behavior compared to the baseline model. The magnitude velocity pathlines for the Land Rover Discovery with roof alteration are seen in Fig. 15c. Near the rear entrance of the automobile, the magnitudes of air velocities overlap. Figure 15d illustrates that the optimal aerodynamic performance is obtained by integrating the ventilation duct and roof alteration. The air velocity is low in two regions: under the car body and inside the duct. It is found the velocity of air at the exit of the duct is higher than at the inlet. Figure 16a,b show the spatial structure of the vortices around the baseline Discovery car from two different views to provide a clear visualization. Vortices behind the car have the greatest effect on the overall behavior. Wheels and wheelhouses generate fairly strong vortices. The front bumper and A-pillar create moderate vortices in this type of car. Figure 16 shows the wake structure for the baseline model using an iso-surface of a constant velocity of air. It illustrates the importance of modifying the back of the car.

**Figure 17:**
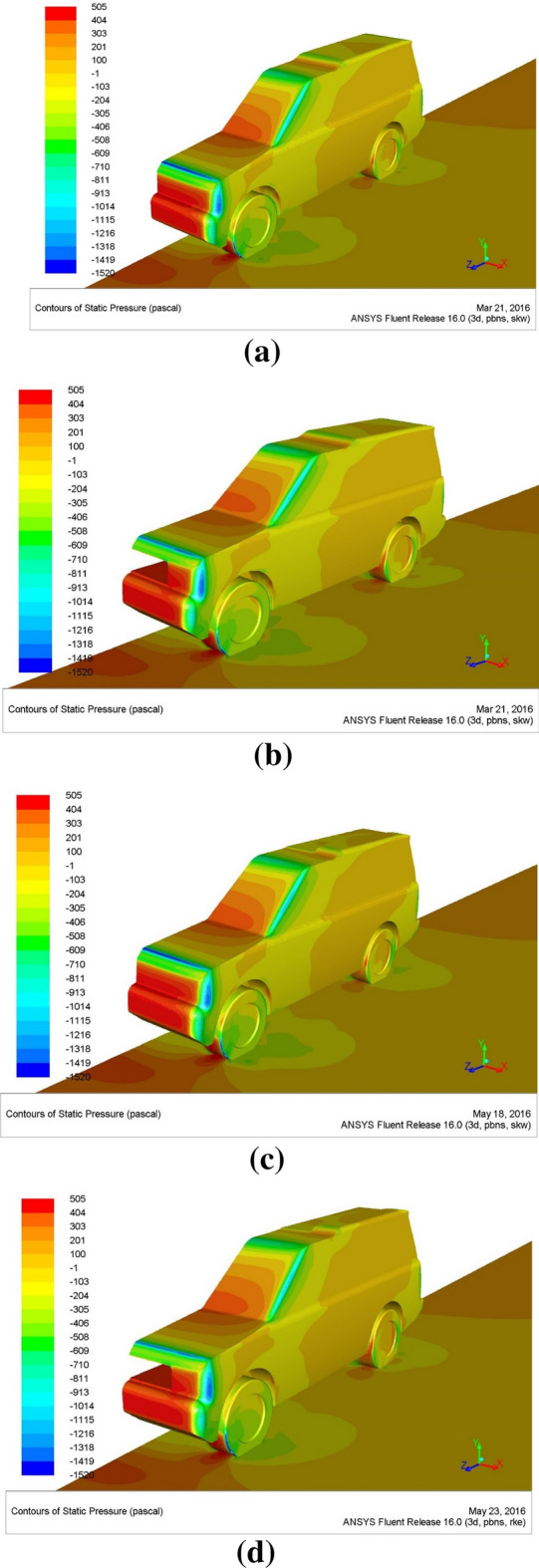
**(a)** Contours of pressure for the baseline of the Land Rover Discovery. **(b)** Contours of pressure for the Land Rover Discovery with the ventilation duct. **(c)** Contours of pressure for the Land Rover Discovery with roof modification. **(d)** The ventilation duct and roof modification contain pressure for the Land Rover Discovery.

**Figure 18:**
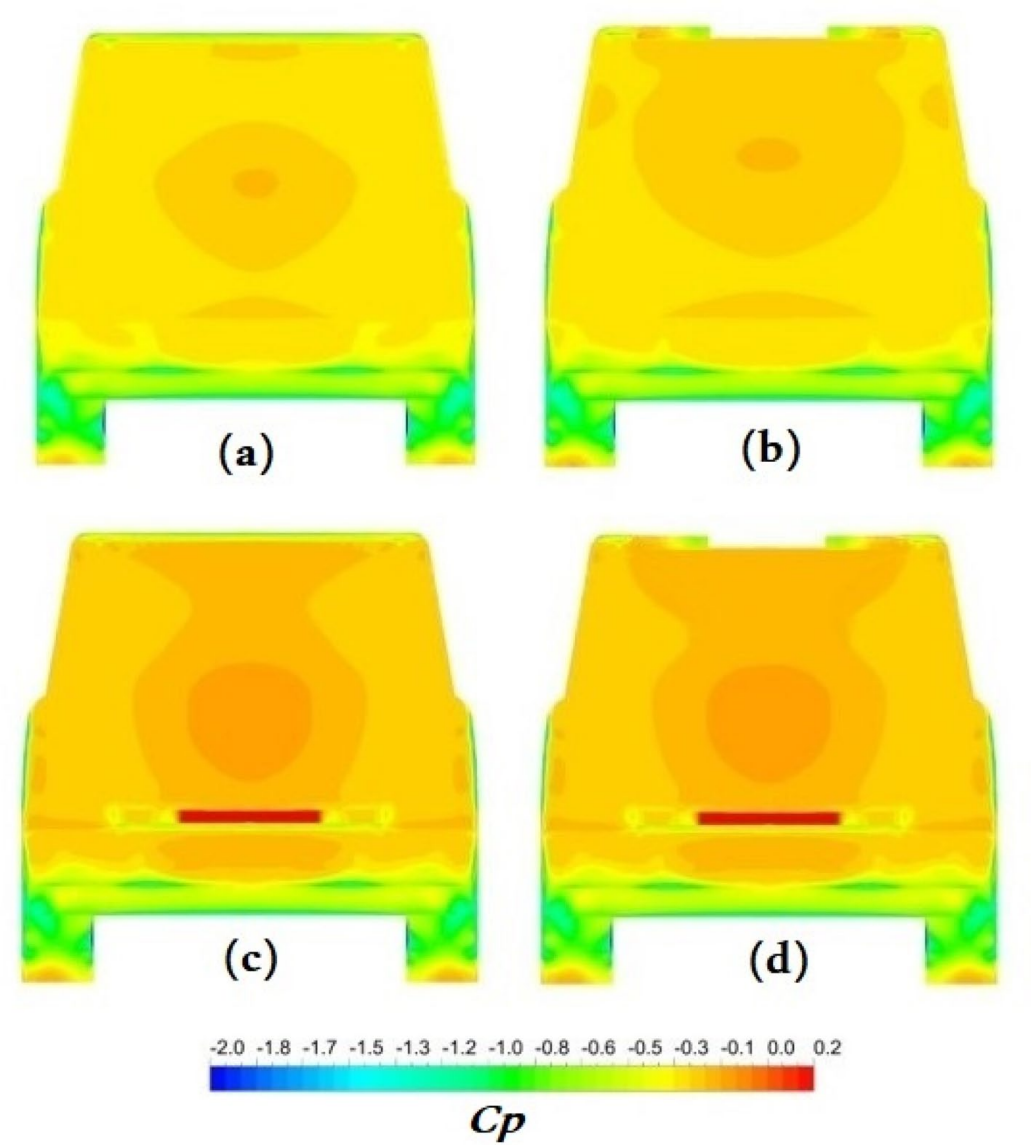
Pressure distribution on the surfaces of the Discovery car from the back view: (**a**) the baseline model, (**b**) the modified roof model, (**c**) the ventilation duct model, (**d**) the combined model.

Figure 17 depicts static pressure contours at an initial air velocity of 28 m/s, plotted on the car’s surfaces and the road. The figures clearly show the variation of the static pressure on the car and over the road. For the baseline model, as illustrated in Fig. 17a, there are some problems with the pressure distribution on the automobile body. The static pressure above the vehicle is acceptable; however, there is a problem with static pressure in front of the roof and the bonnet, which may impact the vehicle’s road balance. The static pressure is found to be rather considerable., particularly on the bumper, lights, and grid of the automobile’s front. This causes more obstructing to the movement of the car. However, by adding the duct, these problems have been reduced, as seen in Fig. 17b. As illustrated in Fig. 17c, the static pressure above the car’s rooftop rises when the car’s rooftop is adjusted, especially near the end of the rooftop. As seen in Fig. 17d, the aerodynamic behavior can be improved by combining the ventilation duct and roof modification. Figure 18 shows the pressure coefficient on the surfaces of the Discovery car from the back view for modified models of the Discovery car as well as the baseline model of it.

Figure 18a depicts the pressure distribution on the Discovery car's surfaces from the back in all configurations. Because of the Discovery's box-like design and alterations in this area, pressure variations are noticeable in the back. Most zones have negative pressures behind the vehicle, resulting in increased opposition. Using a trench on the roof, as illustrated in Fig. 18b, increases pressure along the landing edge and across the back windshield. The vent duct, as illustrated in Fig. 18c, raises the pressure behind the automobile. Coupling a roof ditch with a ventilation duct, as depicted in Fig. 18d, has the potential to boost the pressure behind the Discovery more than other improvements.

**Figure 19:**
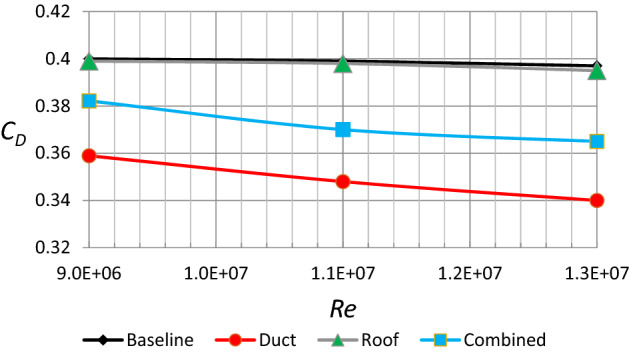
Drag coefficient as a function of Reynolds number for different configurations of Discovery car.

Figure 19 depicts the drag coefficient as a function of Reynolds quantity for a Discovery vehicle baseline and customized versions. To evaluate the link between $Re$ and drag factor, Reynolds quantities ranging from 9 × 10^6^ to 13 × 10^6^ are employed. In general, the drag coefficient falls with rising $Re$ for all Discovery vehicle layouts. A ventilation duct with $Re$ of 13 × 10^6^ achieves the lowest drag factor. As a result, the Discovery automobile with the ventilation duct is regarded as the best-modified type for the drag factor.

**Figure 20:**
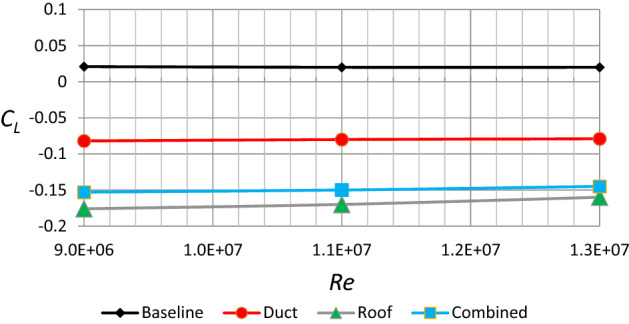
Lift coefficient as a function of Reynolds number for different configurations of Discovery car.

Figure 20 depicts the lift coefficient as a function of $Re$ for all Discovery vehicle variants. The lift coefficient in the baseline model is positive and rises with rising Reynolds quantity. While the lift coefficient in all modified versions is negative and falls with increasing $Re$. Because it provides greater downward force than other types, the modified roof type has the highest lift factor.

Tables 3 and 4 give a brief overview of all the findings. The influence of velocity on drag and lift force can be seen in Table 3 (depending on the realizable *k*–$\varepsilon$ turbulence model). As you might assume, the drag and lift forces rise as the air velocity increases. However, with all modifications, the drag force decreases while the lift force becomes negative, i.e., a car becomes more stable on the road. The drag and lift coefficients for all automobile models are shown in Table 4. As can be seen, the ventilation duct has the lowest drag coefficient, while roof alteration has the lowest lift coefficient. One could conclude the most efficient design for fuel consumption is the use of duct only, as it has resulted in the smallest drag coefficient, while the lift value, although not the lowest, is still negative and acceptable. Nevertheless, the most stable condition combined with the lowest drag coefficient is achieved by both modifications.

**Table 3:** Forces of drag and lift as a function of velocity.

	Velocity ($\mathrm{m}/\mathrm{s}$)	The baseline model		model of a car with a ventilation duct		Model of a car with roof modification		Model of a car with all modifications	
		${F}_{D} (\mathrm{N})$	${F}_{L} (\mathrm{N})$	${F}_{D} (\mathrm{N})$	${F}_{L} (\mathrm{N})$	${F}_{D} (\mathrm{N})$	${F}_{L} (\mathrm{N})$	${F}_{D} (\mathrm{N})$	${F}_{L} (\mathrm{N})$
1	28	578	123	519	−119	573	−253	548	−219
2	34	853	182	765	−176	844	−373	809	−323
3	40	1180	252	1059	−243	1169	−516	1120	−447

**Table 4:** Coefficients of lift and drag for all configurations of the Discovery.

	${C}_{D}$	${C}_{L}$
Baseline model	0.4	0.021
Car model with ventilation duct	0.359	−0.082
Car model with roof modification	0.399	−0.176
Car model with all modifications	0.382	−0.153

## Conclusions

The aerodynamic performance of the Land Rover Discovery was modeled using CFD using numerous modification models to enhance the drag coefficient and the stability of the automobile. Because the system is symmetric, half of the computational domain was used to minimize calculation time. Four turbulence models with the optimum value of elements (between 13 × 10^6^ and 15 × 10^6^) were tested in this investigation. The results of all types of turbulence models (realizable *k*–$\varepsilon$ , standard *k*–$\omega$, shear stress transport $k-\omega$, and Reynolds stress model) were very close to the experimental data, but the Realizable $k-\varepsilon$ result was the most accurate. A ventilation duct’s main purpose is to generate a pressure differential between the two sides of the vehicle, dropping pressure in the front and boosting it in the back, resulting in a reduced drag coefficient. The car’s roof modification puts more pressure above the car, which improves the vehicle’s road stability, especially at high speeds. A combination of the ventilation duct and roof alteration gives better fuel usage efficiency and road safety, with ${C}_{D}$ of 0.382791 compared to the baseline model and ${C}_{L}$ of −0.153046 compared to the baseline model.

## Data Availability

All data generated or analysed during this study are included in this published article.
